# Metagenome techniques to reduce diagnostic delay in Acanthamoeba keratitis

**DOI:** 10.22336/rjo.2021.63

**Published:** 2021

**Authors:** Davide Borroni, Rahul Rachwani-Anil, José María Sánchez González, Marina Rodríguez-Calvo-de-Mora, Carlos Rocha de Lossada

**Affiliations:** * The Veneto Eye Bank Foundation, Venice, Italy; ** Department of Doctoral Studies, Riga Stradins University, Riga, Latvia; *** Department of Ophthalmology, Regional University Hospital of Malaga, Malaga, Spain; **** Department of Physics of Condensed Matter, University of Seville, Seville, Spain; ***** Department of Ophthalmology (Tecnolaser Clinic Vision®), Seville, Spain; ****** Department of Ophthalmology, Regional University Hospital of Malaga, Malaga, Spain; ******* Department of Ophthalmology (Qvision), VITHAS Hospital, Almería, Spain; Department of Ophthalmology, Hospital Virgen de las Nieves, Granada, Spain; Department of Ophthalmology, Ceuta Medical Center, Ceuta, Spain

To the Editor,

We read with interest the article by Shah YS et al. “Delayed diagnoses of Acanthamoeba keratitis at a tertiary care medical center” [**1**]. 

The article well describes difficulties to diagnose microbial keratitis. As reported in **Table 1**, Acanthamoeba keratitis (AK) is usually misdiagnosed with herpetic keratitis [**1**]. We have had same and similar issues [**2**,**3**]. 

Timely diagnosis is the key for an effective treatment. However, the current conventional diagnostic methods (CDM) of stain and culture are time consuming and often with no clinically useful results [**4**]. 

Consequently, efforts need to be done to develop highly sensitive and accurate molecular diagnostic tools with the aim to provide rapid diagnosis and reduce the threat of antimicrobial resistance. 

In recent years, we have started to evaluate the analysis of the metagenome [**5**,**6**].

Metagenomics analysis with next-generation sequencers is becoming more common. The first difficulties to such a new approach improved during time [**7**]. Nowadays, with some sets, it is possible to have results in 24-36 hours [**6**] with reduced costs than in the past [**8**]. 

Additionally, as recently reported by Yu-Jen et al., bacterial 16S ribosomal RNA sequencing techniques have the potential to profile the microbiome in contact lens associated with AK to characterize the association between *Acanthamoeba* and the intracellular microbiome [**9**]. This way, metagenomics analysis will move the investigator from a hypothesis guided approach of CDM to a hypothesis-free approach, with a more comprehensive evaluation of samples [**6**,**10**].

Therefore, we hope that a widespread use of metagenomics analysis could improve the diagnosis of AK and reduce delays.

**Conflict of Interest statement**

Authors state no conflict of interest.

**Acknowledgements**

None.

**Sources of Funding**

None.

**Disclosures**

None.
